# Cassava begomovirus species diversity changes during plant vegetative cycles

**DOI:** 10.3389/fmicb.2023.1163566

**Published:** 2023-05-25

**Authors:** Anna E. Dye, Brenda Muga, Jenniffer Mwangi, J. Steen Hoyer, Vanessa Ly, Yamilex Rosado, William Sharpee, Benard Mware, Mary Wambugu, Paul Labadie, David Deppong, Louis Jackai, Alana Jacobson, George Kennedy, Elijah Ateka, Siobain Duffy, Linda Hanley-Bowdoin, Ignazio Carbone, José Trinidad Ascencio-Ibáñez

**Affiliations:** ^1^Department of Plant and Microbial Biology, North Carolina State University, Raleigh, NC, United States; ^2^Department of Horticulture, Jomo Kenyatta University of Agriculture and Technology, Nairobi, Kenya; ^3^Department of Ecology, Evolution and Natural Resources, Rutgers University, New Brunswick, NJ, United States; ^4^Department of Molecular and Structural Biochemistry, North Carolina State University, Raleigh, NC, United States; ^5^International Livestock Research Institute (ILRI), Nairobi, Kenya; ^6^Department of Entomology and Plant Pathology, North Carolina State University, Raleigh, NC, United States; ^7^Department of Natural Resources and Environmental Design, North Carolina Agricultural and Technical State University, Greensboro, NC, United States; ^8^Department of Entomology and Plant Pathology, Auburn University, Auburn, AL, United States; ^9^Center for Integrated Fungal Research, Department of Entomology and Plant Pathology, North Carolina State University, Raleigh, NC, United States

**Keywords:** plant virus, Cassava (*Manihot esculenta*), vegetative (asexual) propagation, vector transmission, whitefly (*Bemisia tabaci*)

## Abstract

Cassava is a root crop important for global food security and the third biggest source of calories on the African continent. Cassava production is threatened by Cassava mosaic disease (CMD), which is caused by a complex of single-stranded DNA viruses (family: *Geminiviridae*, genus: *Begomovirus*) that are transmitted by the sweet potato whitefly (*Bemisia tabaci*). Understanding the dynamics of different cassava mosaic begomovirus (CMB) species through time is important for contextualizing disease trends. Cassava plants with CMD symptoms were sampled in Lake Victoria and coastal regions of Kenya before transfer to a greenhouse setting and regular propagation. The field-collected and greenhouse samples were sequenced using Illumina short-read sequencing and analyzed on the Galaxy platform. In the field-collected samples, African cassava mosaic virus (ACMV), East African cassava mosaic virus (EACMV), East African cassava mosaic Kenya virus (EACMKV), and East African cassava mosaic virus-Uganda variant (EACMV-Ug) were detected in samples from the Lake Victoria region, while EACMV and East African mosaic Zanzibar virus (EACMZV) were found in the coastal region. Many of the field-collected samples had mixed infections of EACMV and another begomovirus. After 3 years of regrowth in the greenhouse, only EACMV-like viruses were detected in all samples. The results suggest that in these samples, EACMV becomes the dominant virus through vegetative propagation in a greenhouse. This differed from whitefly transmission results. Cassava plants were inoculated with ACMV and another EACMV-like virus, East African cassava mosaic Cameroon virus (EACMCV). Only ACMV was transmitted by whiteflies from these plants to recipient plants, as indicated by sequencing reads and copy number data. These results suggest that whitefly transmission and vegetative transmission lead to different outcomes for ACMV and EACMV-like viruses.

## 1. Introduction

Cassava (*Manihot esculenta* Crantz) is a temperature-resilient and drought-resistant crop that is important to smallholder farmers. Storage roots are harvested for consumption and commercial applications and are important for both food and economic security in Africa. Sub-Saharan Africa produces over half of the cassava grown worldwide (Food Agriculture Organization of the United Nations, 2020). Production of cassava is expected to increase to meet the anticipated decrease in the production of maize and rice as temperature increases (MoALFI, 2019; Ray et al., 2019; Harvesters, 2021). Cassava is a promising crop, but its production is threatened by several viral diseases, including cassava mosaic disease (CMD)—a viral disease that is endemic across Africa and causes major crop losses (Legg et al., 2011; Rey and Vanderschuren, 2017). CMD is characterized by leaf yellowing, deformation, and stunting. These physiological changes impact storage root development and often cause severe reduction in size. Although economic losses caused by CMD have not been calculated on a regional scale since the early 2000s (Legg et al., 2006), recent estimates point to sustained crop losses in both Kenya and other East African countries (Arama et al., 2016; Tembo et al., 2017).

**Figure 1:**
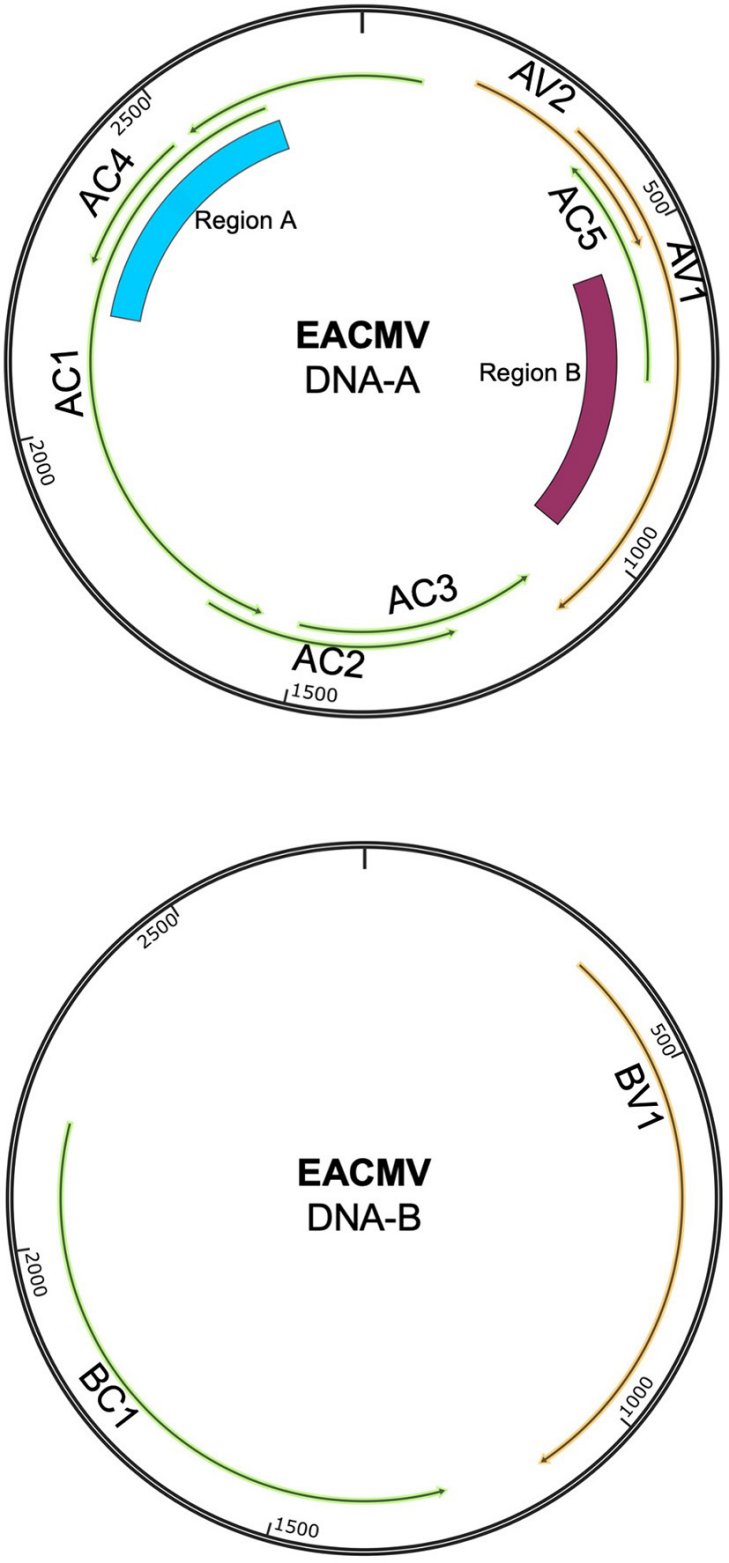
Genome map showing the DNA-A and DNA-B components of EACMV. Viral open-reading frames and genes are marked on both components. The ~400-bp region that distinguishes EACMV-like species is marked as Region A. This region overlaps with the AC4 and AC1 (Rep) open-reading frames. The area of recombination between ACMV and EACMV is marked as Region B. This region overlaps with the open-reading frames for AV1 (CP) and AC5. Genome maps were constructed in SnapGene.

Cassava mosaic disease (CMD) is caused by a complex of single-stranded DNA viruses in the *Begomovirus* genus (family: *Geminiviridae*) (Patil and Fauquet, 2009). Cassava mosaic begomoviruses (CMBs) have bipartite genomes with DNA-A and DNA-B components. Both components are required to establish a systemic infection (Figure 1). The DNA-A component encodes for replication (Rep, REn), encapsidation (CP), and anti-host defense functions (TrAP, AV2, and AC4), whereas the DNA-B component encodes for two movement proteins—the nuclear shuttle protein (NSP) and the movement protein (MP) (Hanley-Bowdoin et al., 2013). The AC5 ORF is hypothesized to have anti-silencing functions (Wu et al., 2022). CMBs are transmitted by whiteflies in the *Bemisia tabaci* cryptic species complex in a persistent, non-propagative manner (Mugerwa et al., 2012). Begomoviruses have high rates of mutation and recombination, leading to intra-host diversity and the emergence of novel species of begomoviruses (Duffy and Holmes, 2008, 2009; Crespo-Bellido et al., 2021; Mishra et al., 2022).

To date, 11 cassava mosaic begomovirus (CMB) species have been identified, with nine of them found in Africa (Jacobson et al., 2018). These species reflect a combination of evolutionary forces including mutation and recombination in mixed infections. Six of the African viruses are known recombinants (Crespo-Bellido et al., 2021). For example, East African cassava mosaic Zanzibar virus (EACMZV) is thought to be a recombinant of East African cassava mosaic Kenya virus (EACMKV) and South African cassava mosaic virus (SACMV), while EACMKV likely originated via a recombination event involving East African cassava mosaic Cameroon virus (EACMCV), which is itself a recombinant of East African cassava mosaic virus (EACMV) and an unknown virus (Crespo-Bellido et al., 2021). Begomoviruses are classified as distinct species when their DNA-A components show < 91% identity (Brown et al., 2015). Because individual CMB DNA-B components often co-infect with highly divergent DNA-A components (reassortant viruses), their sequences are not used for species classification.

African cassava mosaic virus (AMCV) and EACMV are thought to be the ancestral CMB species in sub-Saharan Africa (Jacobson et al., 2018). In the 1990s, a CMD pandemic spread from the Lake Victoria region in Uganda into central and eastern Africa (Legg and Thresh, 2000). The pandemic led to severe cassava crop loss, with up to 100% yield loss. The pandemic was associated with three main factors: (1) a region of the ACMV coat protein (CP) recombined with EACMV DNA-A, resulting in a recombinant viral strain, EACMV-UG; (2) synergistic mixed infection with EACMV-UG and ACMV caused severe disease; and (3) the *B. tabaci* vector became superabundant at the wavefront of the pandemic (Zhou et al., 1997; Pita et al., 2001; Legg et al., 2006). The introduction of virus-resistant cassava cultivars and a reduction in whitefly populations have reduced disease impacts in the region, but CMD is still an important threat to cassava production (Were et al., 2021; Mwebaze et al., 2022). In addition, EACMV-UG continues to spread through Central Africa and toward West Africa, where it could negatively impact the large-scale cassava production in the region (Akinbade et al., 2010; Food Agriculture Organization of the United Nations, 2020; Mouketou et al., 2022).

Cassava mosaic begomoviruses (CMBs) are transmitted by vegetative propagation and whiteflies (Legg et al., 2015). This “vertical” transmission has the potential to transmit a diverse viral population during cycles of vegetative cutting and regrowth, while several filtering barriers in the whitefly body may reduce viral diversity during horizontal transmission. Thus, elucidating the effects of different transmission modes is important for understanding past viral pandemics and predicting future patterns of CMB spread. The exchange of infected cuttings leads to disease spread between farms and regions. It is estimated that 80% of disease transmission in Kenya is due to vegetative propagation (Mwatuni et al., 2015). Given the high frequency of vegetative transmission, it is essential to understand how this agricultural practice impacts changes in viral prevalence and species diversity. A few studies have evaluated the effect of vegetative transmission in other viral systems in potato and sugar cane (Sastry, 2013; Ranawaka et al., 2020). One study in cassava showed that CMB diversity increases through successive rounds of vegetative propagation (Aimone et al., 2021b). This study was performed under laboratory conditions using infectious clones of ACMV and EACMCV as the starting inoculum and, thus, may not fully represent the dynamics in field-infected plants.

The distributions of CMBs across different regions of sub-Saharan Africa have been characterized using field survey data (Ntawuruhunga et al., 2007; Chikoti et al., 2015; Harimalala et al., 2015; Tajebe et al., 2015; Doungous et al., 2022). Multiple studies have characterized the distribution of CMB species in Kenya during and after the pandemic (Sseruwagi et al., 2004; Mwatuni et al., 2015; Koima et al., 2018). Field studies provide important snapshot information, but they do not show how propagation affects viral species over time. In light of the laboratory studies showing that vegetative propagation impacts viral diversity (Aimone et al., 2021b), it is important to ask how vegetative transmission affects viral species presence in field-infected plants. Understanding of the effect of different transmission mechanisms on species presence (or species composition) can help inform management strategies and explain past disease trends.

## 2. Methods

### 2.1. Plant collection, growth, and symptoms

**Figure 2:**
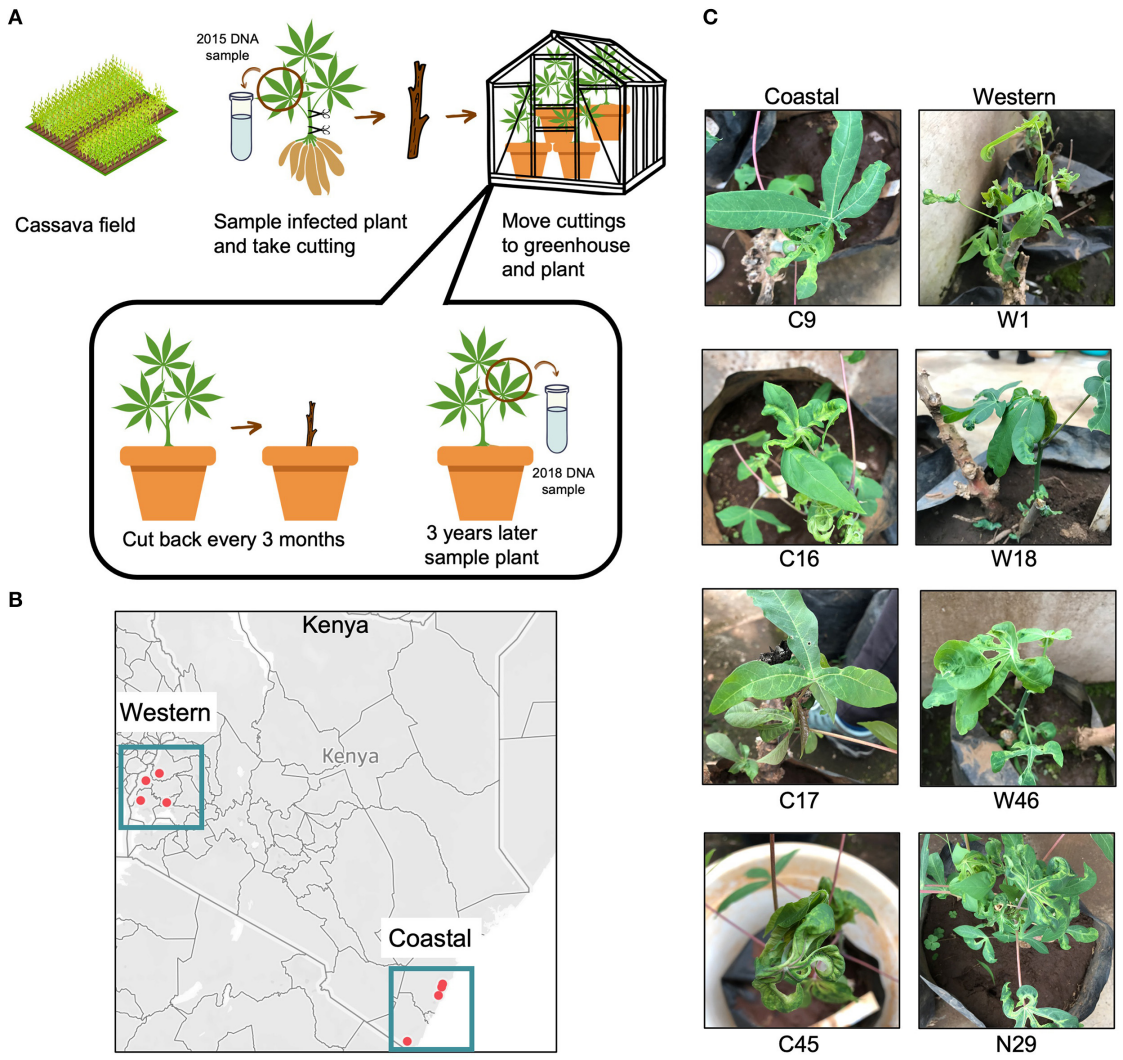
Cassava plants with cassava mosaic disease (CMD) collected from Kenyan fields. **(A)** Workflow showing the experimental design of the vegetative cutting experiment. The workflow was visualized using the Canva platform. **(B)** Points show collection locations of infected cassava in coastal and western areas of Kenya. Two points in the coastal region are close together and appear to overlap but are in two distinct field locations. The map was generated using Tableau. **(C)** Images of the field-collected cassava plants after 3 years of cutting in the greenhouse. Seven of the eight plants displayed severe CMD symptoms, while plant 17C showed no symptoms.

A CMD survey was conducted in coastal (semi-humid to semi-arid) and western (humid) regions of Kenya between June and September 2015. The cassava plants (unknown varieties), in this study, were from different fields separated by at least 10 km in the survey (Sseruwagi et al., 2004). The geo-coordinates (latitude, longitude, and altitude) of each sampling site were recorded using a Global Positioning System (GPS) receiver, GARMIN eTrex Legend (Garmin Ltd, Olathe, KS, USA). The plants were scored for CMD symptom severity at 3–6 months after field planting using a scale of 1 (no symptoms) to 5 (very severe symptoms) (Hahn et al., 1980). At the same time, lower shoot cuttings taken were propagated in an insect-proof greenhouse. The plants were watered daily, and pests were controlled by spraying at 2-week intervals using a broad-spectrum insecticide/miticide (Dynamec 1.8EC, Syngenta), as directed by the manufacturer to control mites, whiteflies, and mealy bugs. The plants were maintained in the greenhouse from 2015 to 2018 by cutting back at 3-month intervals, leaving two active buds for regrowth (Figure 2A). After 3 years of cutting and regrowth (10–13 cycles), the plants were photographed to record symptoms.

### 2.2. DNA isolation and PCR detection of CMBs

In 2015, total nucleic acid was isolated from the uppermost fully expanded leaves of the plants after 2 months in the greenhouse using a modified cetyltrimethylammonium bromide protocol (Lodhi et al., 1994). The 2015 samples were analyzed by PCR to confirm the presence of CMBs shortly after field collection (data not shown) and stored at −20°C. In 2018, after 3 years of repeated cycles of cutting and regrowth, total DNA was extracted from the third visible leaf relative to the apex of each plant using a Qiagen DNeasy Plant kit (Qiagen, Valencia, CA, USA).

Circular viral sequences in the 2015 and 2018 samples were subjected to linear amplification using an EquiPhi29 kit (Thermo Fisher Scientific, Waltham, MA), with 2 μl of total DNA as the input for rolling circle amplification (RCA) (Haible et al., 2006; Schubert et al., 2007; Jeske, 2009; Johne et al., 2009; Yang et al., 2014; Kathurima et al., 2016). PCR amplification of the RCA products was performed using begomovirus degenerate primers (RepMot: 5′GAGTCTAGAGGATANGTRAGGAAATARTTCTTGGC3′ and CPMot: 5′CGCGAATTCGACTGGACCTTACATGGNCCTTCAC3′) (Ascencio-Ibanez et al., 2002) and HotStart Taq Polymerase (Qiagen, Valencia, CA). The reactions were performed for 35 cycles (denaturation: 30 s at 95°C, annealing: 1 min at 54°C, and elongation: 30 s at 72°C). Field- (2015) and greenhouse-collected (2018) samples were tested for the presence of begomoviruses. Four plants from the Lake Victoria region and four plants from the coastal region were selected for Illumina sequencing using the criteria that both the field and greenhouse samples for that plant tested positive for begomoviruses by PCR. One greenhouse sample did not test positive for begomoviruses (C17), but it was included to increase the number of samples for the coastal region. The selected samples were used for deep sequencing.

### 2.3. Library preparation and sequencing

Sequencing libraries were prepared using a method to enhance viral read counts for ssDNA virus genomes (Aimone et al., 2022). Two RCA reactions were performed for each sample using 2 μl of input DNA and the EquiPhi29 kit. After RCA, the products were end-repaired using Klenow and T4 DNA polymerase (New England BioLabs Inc., Ipswich, MA, USA), purified using 1.2 × SPRI select beads (Beckman Coulter, Chaska, Minnesota, USA), and quantified using a Qubit fluorometer (Invitrogen, Waltham, Massachusetts). The RCA products from the two reactions were pooled after normalization of their concentrations and used to generate two libraries using an Illumina Nextera XT kit and IDT Unique Dual Indexes selection (Illumina, Inc., San Diego, California, USA; Integrated DNA Technologies, Coralville, IA, USA). The libraries were cleaned up using 0.8× SPRI select beads to remove library constructs with small inserts, normalized to 10 nM/library, and pooled for sequencing. Paired-end 150-bp reads were generated using the Illumina NovaSeq 6000 S4 platform for the eight sample pairs (field and greenhouse) with two technical replicates each for a total of 32 libraries.

### 2.4. Processing reads for reference-guided and *de novo* assemblies

**Figure 3:**
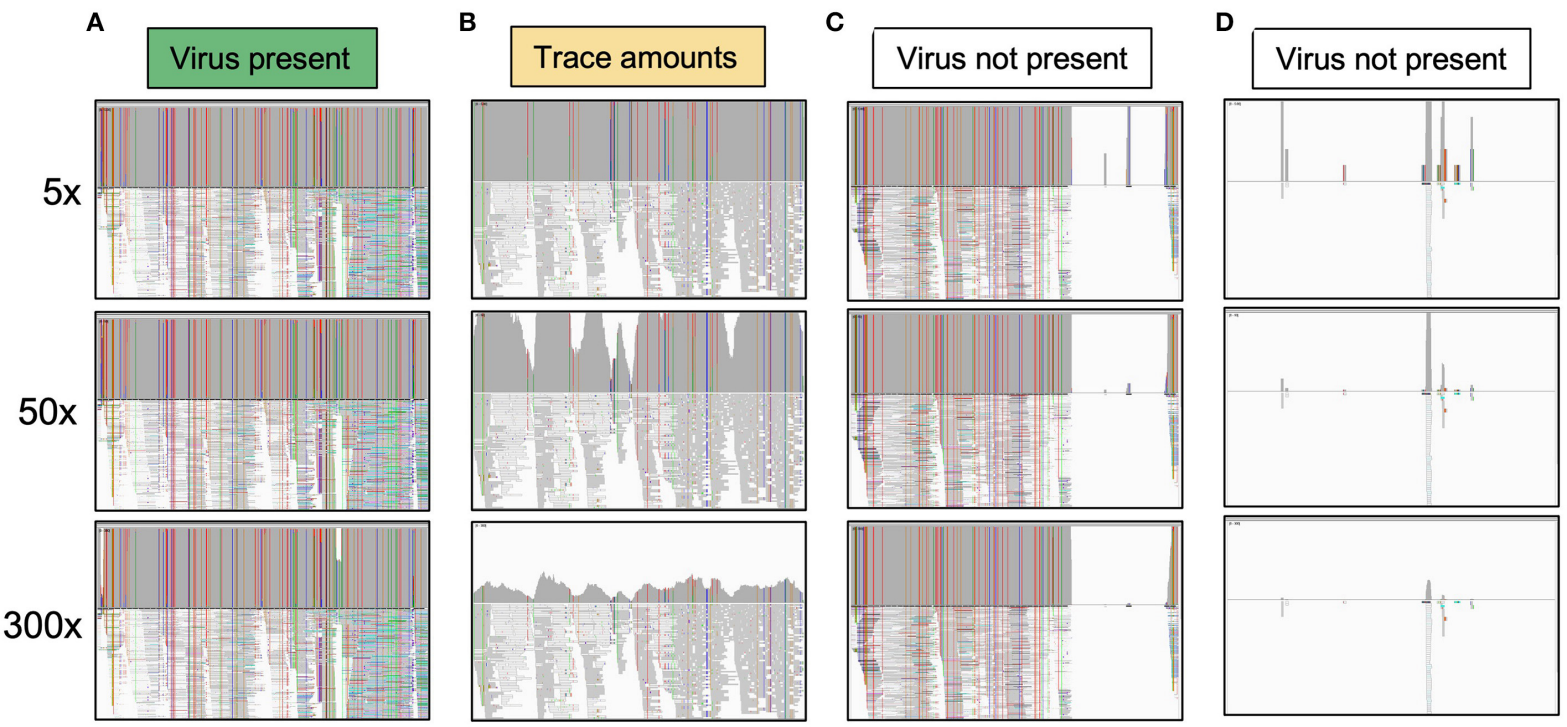
System for classifying the presence of viral species in a sample based on read coverage. Each category shows the IGV visualization of coverage across a viral genome component. **(A)** The virus is marked as present when it reaches 50× coverage. All viruses counted as present had at least 150× coverage, and all but one had >300× coverage. **(B)** An example of a virus present in trace amounts. The virus reaches the 5× threshold across over 95% of the genome component but does not meet the threshold for 50× coverage. **(C)** This example shows a virus that has high coverage for part of a genome component but not the entire genome component, indicating that the viral species queried is not present. Likely a related virus is present that shares some sequence identity for part of the genome. **(D)** This example shows that the virus is clearly not present. At any of the thresholds, the read coverage is not met across the genome component.

Sequencing reads were processed using the ViralSeqMapping Pipeline on the Galaxy platform (Afgan et al., 2018; Aimone et al., 2022) (ViralSeq: https://cassavavirusevolution.vcl.ncsu.edu/). Sequencing adapters were trimmed, low-quality reads were discarded using Cutadapt (Martin, 2011), and reads were mapped to viral genomes using BWA-MEM (Li and Durbin, 2009; Li, 2013). Reads were mapped to the following reference sequences: EACMCV (monomer units in GenBank accessions MT856195.1 and MT856192.1), ACMV (MT858793.1 and MT858794.1), EACMV (MZ570970.1 and MZ570971.1), EACMV-UG (MK059418.1), EACMKV (AJ717572.1 and AJ704971.1), SACMV (AF155806.1 and AF155807.2), and EACMZV (AF422174.1 and AF422175.2). Reference sequences were chosen based on the CMBs previously recorded in Kenya and two CMBs (EACMCV and SACMV) that have not been recorded in Kenya as background controls. Mapped reads were sorted by coordinate order using SortSam in Picard tools (https://broadinstitute.github.io/picard/), PCR duplicate reads were removed using Picard MarkDuplicates, and read coverage was visualized using IGV (Robinson et al., 2011). Genomes were categorized as present with high amounts of virus in the sample, present with trace amounts of virus in the sample, or absent without detectable virus in the sample (Figure 3). Categories were determined by a consistent threshold of coverage across at least 95% of the DNA-A component for each species. 300× coverage was the threshold for high presence, 5× coverage was chosen as the threshold for trace amounts, and all samples below 5× coverage were treated as absent. Because of the recombinant nature of EACMV-like viruses, a 400-bp region that showed a high difference between EACMV, EACMKV, and EACMV was used to determine which EACMV-like virus was present.

*De novo* contigs were assembled from trimmed reads with MEGAHIT (Li et al., 2015), with the following arguments: –k-min 31 –min-count 3 –min-contig-len 500 –no-mercy. Contigs with begomovirus sequence similarity were identified using NCBI BLAST+ blastn on the Galaxy platform, with the reference sequences listed above as the subject sequences. The megablast setting was used, and the expectation value cutoff was set to 0.001. The contigs were analyzed further by querying the sequences using megablast against the entire BLAST database on the NCBI BLAST database (Sayers et al., 2021).

### 2.5. Consensus sequences

For each library, consensus sequences were generated using the “GetConsensus” pipeline on the Galaxy platform (GetConsensus: https://cassavavirusevolution.vcl.ncsu.edu/), which mapped reads to viruses present in the selected sample using BWA-MEM (Li and Durbin, 2009). Variants were called using Samtools mpileup and Varscan (Koboldt et al., 2012). Varscan was set to only include variants with over 50% prevalence in the sample and to include both indels and SNPs (Koboldt et al., 2012). The majority of variants were, then, used to create a consensus sequence using bcftools consensus. The pipeline ran the reads through mapping, mpileup generation, and variant calling in three successive rounds to ensure that all reads were appropriately mapped and checked for accuracy. Consensus sequences for individual viral components were generated for each technical replicate from both field- and greenhouse-collected samples. Consensus sequences for each component were aligned using MAFFT (Katoh and Standley, 2013) and implemented in the DeCIFR portal (https://tools.cifr.ncsu.edu/mafft). Multiple sequence alignments were visualized and compared for the similarity between replicates using the SnapGene software (Insightful Science; snapgene.com). Multiple sequence alignments were visualized with SnapGene software (Insightful Science; snapgene.com). Because of the levels of similarity between the technical replicates, consensus sequences from one replicate from each sample were used for subsequent analyses. Consensus sequences are available in Supplementary material “S1. Consensus sequences.”

### 2.6. Phylogenetic trees

Consensus sequences were compiled into loci files and used to generate phylogenetic trees with the *de novo* tree inference tool (https://tools.cifr.ncsu.edu/denovo) in DeCIFR. The best-scoring maximum likelihood tree was based on 500 bootstrap replicates, and a GTRGAMMA model of evolution was performed using RAxML v8 (Stamatakis, 2014). Phylogenetic trees were inferred for each viral component separately. Trees were visualized using T-BAS v2.3 (https://tbas.cifr.ncsu.edu/) (Carbone et al., 2017, 2019).

### 2.7. Whitefly transmission

Cassava plants (cv. Kibandameno and cv. 60444) were co-inoculated with EACMCV (MT856195.1 and MT856192.1) (Fondong et al., 2000; Fondong and Chen, 2011; Hoyer et al., 2020) and ACMV (MT856193.2 and MT856194.1) infectious clone DNA (AddGene plasmids 159134 to 159137) by low-pressure biolistic bombardment (Aimone et al., 2022). ACMV and EACMCV produced in plants infected using viral clones can be acquired by whiteflies and subsequently transmitted into sucrose substrates and leaf disks (Kennedy et al., 2023). Three symptomatic cassava plants (two 60444 and one Kibandameno) were used as sources for three bioreplicates of whitefly transmission. One recipient plant per experiment was sequenced with the exception of the T8 lineage, for which two recipient plants were sequenced. The plants were placed in three separate insect cages with 400 non-viruliferous whiteflies for a 48-h acquisition access period (AAP).

Whiteflies were obtained from a colony of *B. tabaci* initiated from the offspring of 20 adults collected in 2016 from cassava fields in Kisumu county, Kenya, as described in the study by Kennedy et al. (2023). All founding adult whiteflies belonged to the SSA1-SG1 clade as determined by amplification of mtCOI using universal primers C1-2195 and L2-N-3014 (Simon et al., 1994) and published protocols (Boykin and De Barro, 2014). The colony was initially reared for at least two generations on eggplant (*Solanum melongena*), a non-host of CMBs, after which it was maintained on virus-free cassava (cv. Kibandameno) plants started in tissue culture.

Non-infected plants were moved into the insect cages to replace the infected plant for a 48-h inoculation access period (IAP) in complete darkness. At the end of the IAP, whiteflies were stored in 70% ethanol, and the recipient plants were treated with imidacloprid insecticide (Admire^®^ Pro, Bayer CropScience). Leaf samples from the top three leaves were taken 28 days after the completion of the IAP. The sampled leaves emerged after whitefly feeding had finished. Total DNA was extracted using a Qiagen DNeasy Plant Mini Kit (Qiagen, Valencia, CA). Viral titers were quantified using quantitative PCR as previously described (Rajabu et al., 2018; Aimone et al., 2022). Samples were amplified in triplicate and compared with a standard curve. Titers are reported as viral copy number/ng of total DNA. DNA libraries were made for each source plant and recipient plant according to the method described above.

## 3. Results

### 3.1. Infected plant sample collection

Leaf samples and stem cuttings were collected from four areas of Kenya in 2015, in western and coastal Kenya (Figure 2B), and GPS locations were recorded as part of a larger study conducted by the Ateka research group. The 2015 DNA samples from the field-collected plants were retested in 2018 in end-point PCR assays using degenerate PCR primers. The expected 750-bp band was amplified from all eight samples, confirming the presence of CMB DNA-A at the time of field collection (Supplementary Figure S1A).

After 3 years of cutting back the plants every 3 months, leaf samples were collected from the eight plants again in 2018. The samples were tested for CMBs using degenerate begomovirus primers. In total, seven of the eight greenhouse samples tested positive for the presence of CMB DNA (Supplementary Figure S1B). The same seven plants exhibited leaf curling and mosaic patterning characteristic of CMD (Figure 2C). In contrast, coastal plant 17C showed no CMD symptoms and contained no detectable levels of CMB DNA-A in PCR assays. These results established that seven of the eight plants propagated for 3 years in the greenhouse were infected with at least one CMB, and only one plant, C17, appeared to have recovered from the infection.

### 3.2. Reference-guided viral genome assembly

Total DNA was used for next-generation sequencing of the eight 2015 and eight 2018 samples. Technical replicates were sequenced to address potential variability in the RCA and PCR amplification steps during library preparation (Aimone et al., 2022). The sequencing reads were mapped against CMB genomes and the cassava genome (NCBI assembly GCA_020916425.1), and the mapped reads from the technical libraries were combined. The mapping statistics are shown in Supplementary Table S1.

Viral genomes were assembled through reference-guided assembly of the sequencing reads. The reference genomes included EACMV, ACMV, EACMV-Ug, EACMKV, and EACMZV, all of which have been documented in Kenya (Mwatuni et al., 2015). The highest coverage was 9,000× for an EACMV-like virus and 4,500× for ACMV. We detected 23 virus instances using a threshold set at 300× coverage across at least 95% of the genome component (Figure 3A). Only one additional virus instance was detected when the coverage threshold was reduced to 50× (This instance was also detected at a 150× threshold). We assigned these 24 virus instances to the ‘present' category (green cells in Table 1). We reduced the threshold to 10× and 5× coverage across 95% of the genome component to detect viruses that occur in trace amounts. The 10× and 5× thresholds uncovered one and eight additional instances, respectively. We assigned these nine instances that are not in the present category to the “trace” category (yellow cells in Table 1) (Figure 3B). The present and trace categories, which distinguish viruses occurring at high coverage vs. low coverage, were used to uncover changes in viral abundance between the 2015 and 2018 time points.

**Table 1:** Viral read counts by sample from reference-guided assembly.

**Area**	**Sample**	**Year**	**EACMV-like A**	**EACMV-like B**	**ACMV-A**	**ACMV-B**
Coastal	C9	2015	101,627	106,795	208	100
		2018	1,256,012	465,484	92	42
	C16	2015	4,093,326	1,437,547	252	16
		2018	2,630,326	1,312,788	190	25
	C17	2015	4,330,732	1,734,657	394	30
		2018	5,531	1,132	0	0
	C45	2015	566,396	723,874	85	70
		2018	1,425,473	1,255,448	154	94
Western	W1	2015	9,039	5,132	326,607	52,980
		2018	3,491,183	3,048,044	1,155	470
	W18	2015	318,034	49,130	37,472	98,751
		2018	1,833,768	1,818,883	81	73
	W46	2015	3,750,519	1,546,347	411,696	453,304
		2018	1,881,391	2,420,126	72	44
	N29	2015	10,541	6,859	43,924	90,385
		2018	2,800,890	3,178,637	921	306

Green shows samples with consistent 300× coverage, indicating that the virus is present.

Yellow shows samples with coverage across the genome that does not reach 300× coverage, indicating that the virus could be present.

Cells with no highlighting show samples without consistent coverage across the genome, even at a 5× threshold, indicating that the virus is not present.

If reads localized to some parts of a viral genome component but were absent in other parts, the virus was not called present even if there were high numbers of mapping reads (white cells in Table 1). Incomplete coverage across the genome component was observed primarily for recombinant viruses with DNA-A components that derived in part from EACMV (Figure 3C). This is illustrated for EACMV-UG, which is a recombinant between EACMV and ACMV and has a gap in read coverage in the AV1 gene of EACMV (Supplementary Figure S2) that matches ACMV AV1 reads. We also mapped the reads to SACMV and EACMCV but only detected mapping to regions shared by other CMBs and not across their entire genomes (data not shown), indicating that these viruses were not present in the Kenyan field samples. When few to no reads mapped to the viral component, it indicated that the virus was not present (Figure 3D).

We initially screened the samples for ACMV and EACMV-like viruses. Because EACMV-like viruses display high sequence similarity across large sections of their genomes, reads mapping to either their DNA-A or DNA-B components were combined (Table 1). All samples at both time points tested positive for EACMV-like DNA-A and DNA-B. The western samples were also positive for ACMV in the 2015 samples but not in the corresponding 2018 samples, indicating that ACMV could only be detected at trace levels after 3 years of greenhouse propagation. None of the plants taken from coastal fields had ACMV at either the 2015 or 2018 time points.

The EACMV-like species were distinguished by mapping to the unique sequence regions of each virus species (Table 2). Samples were also required to have continuous 300× coverage across the entire genome to be called positive for a given EACMV-like virus. EACMV, EACMZV, and EACMKV differ in a ca. 400-bp segment overlapping the AC4 and AC1 genes. All the 2015 coastal samples had >300× coverage of EACMV DNA-A and DNA-B. Three of the coastal samples (9C, 16C, and 45C) were positive for EACMV in 2018. Coastal sample 16C also had >300× for EACMZV in 2015, indicating that the plant was co-infected with EACMV and EACMZV, but we did not detect EACMZV in the 2018 16C sample. The 2018 17C sample had low read counts but consistent coverage for EACMV and EACMZV in 2018, suggesting that the viruses were present at very low levels. EACMKV was not detected in any coastal samples.

**Table 2:** Viral reads mapping specifically to the 400 bp of difference between DNA-A components of EACMV, EACMKV, and EACMZV.

**Area**	**Sample**	**Year**	**EACMV**	**EACMKV**	**EACMZV**
Coast	9C	2015	15,855	4	16
		2018	1,126,388	7	28
	16C	2015	3,170,131	5	1,330,365
		2018	5,609,983	32	20
	17C	2015	3,364,091	6	62
		2018	179	36	588
	45C	2015	167,261	0	20
		2018	648,158	45	23
West	W1	2015	43	326	2
		2018	3,107,571	33	150
	W18	2015	58,464	85	81
		2018	876,022	41,021	13
	W46	2015	3,628,008	89	128
		2018	920,244	50,710	28
	29N	2015	1,222	0	3
		2018	1,240,786	27,938	45

Green shows samples in which the selected virus is present.

Yellow shows samples in which trace amounts of the selected virus are present.

Cells with no highlighting show samples without consistent coverage across the genome, even at a 5× threshold, indicating that the virus is not present.

The EACMV patterns were more varied in the western samples. Two western samples (W18 and W46) were positive for EACMV in both the 2015 and 2018 time points. EACMV was not detected in the 2015 W1 sample but was present in the corresponding 2018 sample. EACMV was detected but did not reach the >300× coverage threshold in the 2015 N29 sample but met the threshold in 2018. EACMKV was detected in two 2015 western samples (W18 and W1), both below the 300× threshold, but reached the 300× threshold in three 2018 samples (W18, W46, and 29N). EACMKV was detected at the 5× coverage threshold in the 2015 W18 sample but reached the 300× threshold in corresponding 2018 sample. In contrast, EACMKV was also detected below the 300× threshold in the 2015 W1 sample and was not detected in the 2018 W1 sample. EACMZV was only detected in trace amounts in W46-2015.

The EACMV-Ug pandemic variant was distinguished from other EACMV-like viruses using a 465-bp region overlapping the AV1 gene that was derived from ACMV by recombination. No coastal samples showed evidence of the presence of EACMV-Ug at either time point. In contrast, EACMV-Ug was detected at >300× coverage in three of the four western samples. Two 2018 western samples (W1 and 29N) also showed trace coverage for EACMV-Ug. However, because ACMV is present in all the samples where the detection of EACMV-Ug was positive, reads from ACMV could have been binned and counted as EACMV-Ug. Similarly, given that there is a similarity between EACMV and EACMV-Ug over most of the genome, an EACMV infection might map to a large portion of the EACMV-Ug reference genome. To address this possibility, coverage across the full DNA segment was viewed in IGV for EACMV and EACMV-Ug (Supplementary Figure S2). Samples that showed a gap in coverage at the EACMV AV1 but full coverage for EACMV-Ug were counted as positive for EACMV-Ug. This analysis indicated that only the 2015 W18, W46, and 29N and 2018 N29 samples were positive for the EACMV-Ug variant, and that the 2018 W1 sample was infected with EACMV and not the Uganda variant (Table 3).

**Table 3:** EACMV and recombinant EACMV-Ug.

**Area**	**Sample**	**Year**	**EACMV**	**EACMV-Ug**
Coast	9C	2015	19,285	16
		2018	208,399	108
	16C	2015	385,307	4
		2018	474,853	9
	17C	2015	324,008	73
		2018	1,066	0
	45C	2015	81,267	2
		2018	174,389	12
West	W1	2015	539	54,707
		2018	373,635	194
	W18	2015	576	59,946
		2018	211,792	0
	29N	2015	983	7,637
		2018	268,011	163
	W46	2015	816	216,159
		2018	195,366	2

Viral reads mapping to the recombinant region of ACMV and EACMV that defines the EACMV-Ug pandemic variant to distinguish which viruses are present. The green color shows samples with consistent 300× coverage, indicating that the virus is present. The coverage must cross the recombination points of the whole-genome component. The yellow color shows samples with coverage across the genome that does not reach the sufficient 300× coverage, indicating that trace amounts of virus are present. The coverage must cross the recombination points of the whole-genome component.

Green shows samples in which the selected virus is present.

Yellow shows samples in which trace amounts of the selected virus are present.

Cells with no highlighting show samples without consistent coverage across the genome, even at a 5× threshold, indicating that the virus is not present.

### 3.3. *De novo* genome assembly

*De novo* genome assemblies were constructed as an alternative to confirm species identified using reference-guided assembly. Full-length and partial-length assemblies were constructed (Table 4). Only the DNA-A components are described in the table because their sequence determines species identity. Full-length *de novo* assemblies are denoted with “F,” and partial assemblies are denoted with “P.” The number of each assembly is noted in parentheses. If more than 35 contigs with similarity to begomoviruses were assembled from a sample, the first 35 contigs were used for the analysis. If <35 contigs were assembled from a sample, all the contigs were used for the analysis. All *de novo* assemblies were required to contain sequences flanking at least one known recombination junction to ensure accurate identification. The table is color-coded green and yellow for comparison to the results obtained using reference-guided assembly. The *de novo* and reference-guided assemblies gave very similar results. *De novo* assemblies detected the same viral components identified as present in the reference-guided assembly in 23 of 24 instances (Table 4, green cells) and trace amounts in four out of nine instances (yellow cells). *De novo* sequences were identified when the reference-guided assembly was not present in four instances. *De novo* but not reference-guided assembly also detected EACMZV in the 2018 C16 sample, but in this case, EACMZV was detected in 2015 by both methods. The 2015 W18 sample was the only sample for which *de novo* assembly detected a DNA-A component (EACMZV) that was not identified by reference-guided assembly in 2015 or by either method in 2018.

**Table 4:** Viral species presence by *de novo* assemblies.

**Area**	**Sample**	**Year**	**EACMV-A**	**EACMKV-A**	**EACMV-Ug-A**	**EACMZV-A**	**ACMV-A**
Coast	C9	2015	F (1)				
		2018	F (1) P (14)				
	C16	2015	P (3)			P (6)	
		2018	P (2)			P (6)	
	C17	2015	F (1) P (11)			P (4)	
		2018	P (2)			F (1) P(1)	
	C45	2015	F (1) P(7)				
		2018	P (10)				
West	W1	2015		F (1)			F (1)
		2018	F (1) P (1)				F (1)
	W18	2015	F (1) P (3)		P (7)	P (2)	F (2) P (3)
		2018	F (2) P (5)	F (1)			
	W46	2015		P (1)	P (8)		P (12)
		2018	F (1) P (7)	P (3)			
	N29	2015	P (7)		P (3)		P (2)
		2018	F (1) P (3)	P (1)			

Green shows which component was marked as present via reference-guided assembly. Yellow shows samples which showed a possible positive for the viral segment via reference-guided assembly. Full and partial *de novo* contigs were assembled.

F, full length *de novo* assembly; P, partial length *de novo* assembly; (x), the number of contigs.

Green shows samples in which the selected virus is present.

Yellow shows samples in which trace amounts of the selected virus is present.

Cells with no highlighting show samples without consistent coverage across the genome, even at a 5× threshold, indicating that the virus is not present.

**Figure 4:**
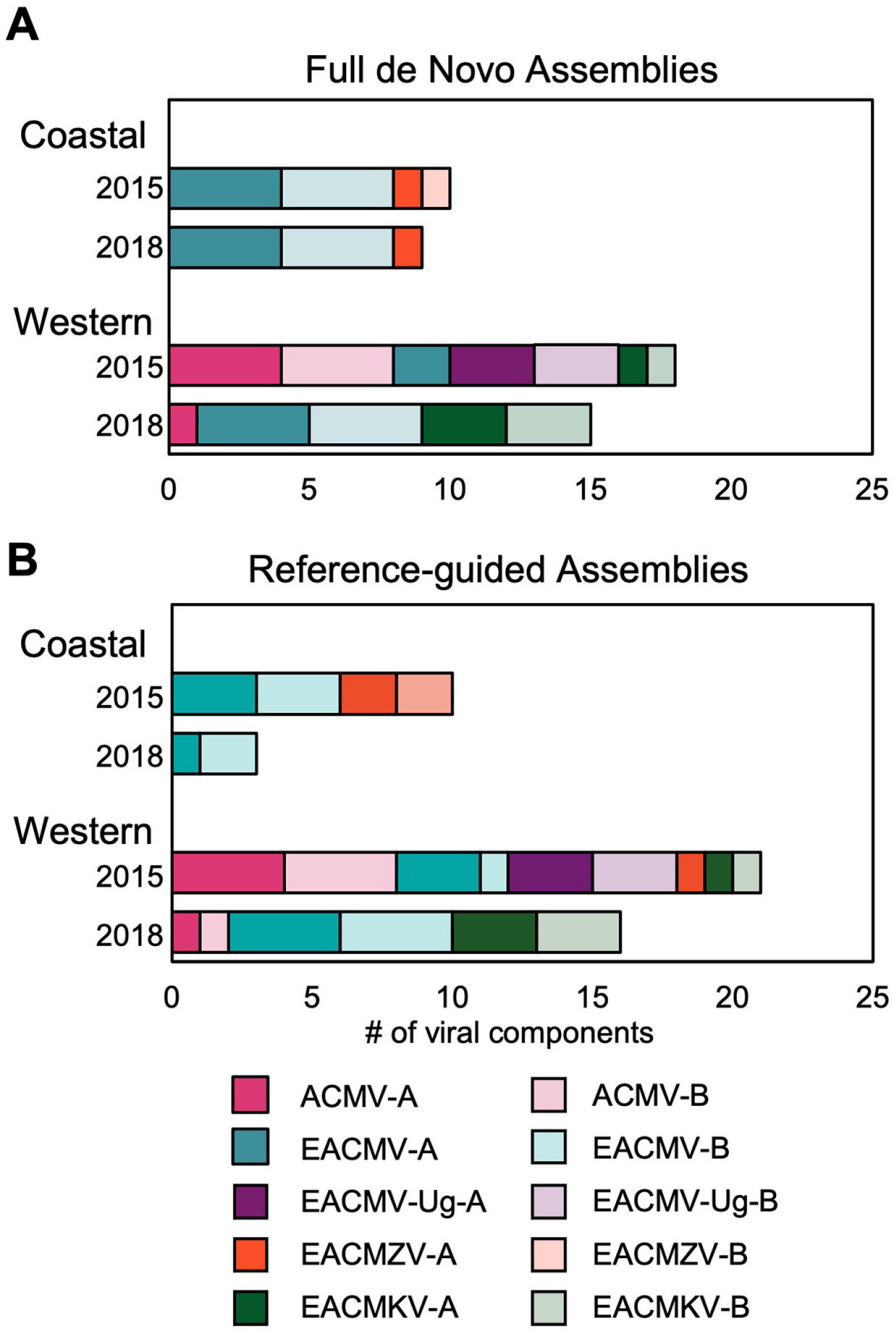
CMB sequence assemblies by region and year. **(A)** Full-length *de novo* genome assemblies generated with MEGAHIT are shown for the DNA-A and DNA-B components for ACMV, EACMV, EACMV-Ug, EACMZV, and EACMKV. The width for each component segment indicates the number of plants in that time point and region that have full-length *de novo* sequences. Wider segments correspond to more samples for the given component. **(B)** The number of viral components identified by the reference-guided assembly is shown using the same width parameters as in panel **(A)**.

We compared the number of samples by time and region that were positive for viral genome components by *de novo* and reference-guided assemblies (Figure 4). This analysis included both DNA-A and DNA-B components. Each bar shows the sample count of the different components with a longer bar indicating more samples in the category. The two methods gave similar results. Both methods detected more CMB species in the 2015 western samples than in the 2015 coastal samples. A decrease in the number of the different viral components was seen in the 2018 samples compared with the 2015 samples for both geographical regions, most likely reflecting a decrease in co-infections and a decline in viral species diversity over time. There is also evidence of reassortment between DNA-A and DNA-B components of different CMBs. For example, EACMV-A, EACMV-B, and EACMZV-A were present in coastal samples at both time points, but EACMZV-B was only in the 2015 samples, suggesting that EACMV-B is providing movement functions for EACMZV-A in 2018 (Bull et al., 2007; Briddon et al., 2010).

Viral consensus sequences were used to generate phylogenetic trees. Sequences were nearly identical between technical replicates for all viral components, with 43/50 sequences sharing >99.95% sequence identity (Supplementary Table S3). A maximum likelihood tree of EACMV DNA-A sequences showed that the Western samples grouped together both in 2015 and 2018 but showed no significant relationship between samples from the same plant for EACMV DNA-A alone (Supplementary Figure S3A). There were substantial numbers of SNPs between 2015 and 2018 EACMV DNA-A consensus sequences from the same plant (ranging from 71 to 105), so we deemphasized studying sequences as sample pairs and consider our data “collected from the field 2015” and “greenhouse data from 2018.” We did not find strong evidence of horizontal transfer of sequences among the greenhouse plants. The only possible exception was W1, which did not have any detectable EACMV in 2015 and had an EACMV DNA-A sequence closely related to the W18 sequence in 2018. Phylogenetic trees of more limited data sets (EACMKV DNA-A and EACMZV DNA-A) also showed no close relationships between 2015 and 2018 samples from the same fields and no phylogenetic relationships indicated horizontal transfer from one plant to another (Supplementary Figure S3B).

### 3.4. Whitefly transmission of CMBs

**Figure 5:**
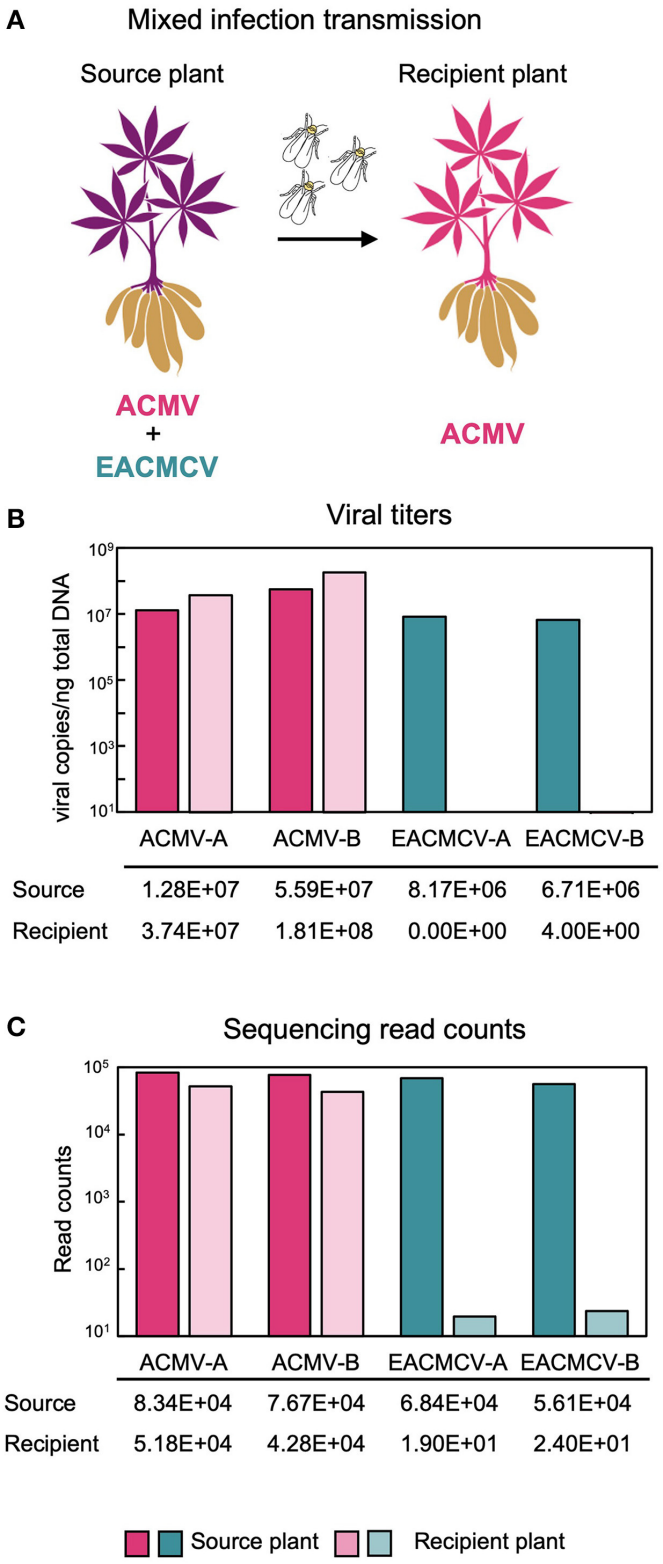
Whitefly transmission of ACMV and EACMCV. **(A)** Diagram of whitefly transmission showing that ACMV but not EACMCV is transmitted by whiteflies from cassava plants co-infected with both viruses in the laboratory. The diagram was generated using the Canva platform. **(B)** Average DNA copy numbers of ACMV and EACMCV in the source (three samples) and recipient (seven samples) plants. **(C)** Average NGS read counts by viral component from the same source and recipient plants.

Our studies showed that when plants started with a co-infection consisting of ACMV and EACMV-like virus, only the EACMV-like virus was present at high levels after 3 years of vegetative cutting. Given that CMBs are also transmitted by silverleaf whiteflies, we asked if vector transmission of ACMV and EACMV-like viruses also shows a bias. For these studies, we used ACMV and East African cassava mosaic Cameroon virus (EACMCV) for whitefly transmission studies in a controlled environment (Figure 5A). EACMCV is likely a recombinant virus of EACMV and an unknown virus (Fondong et al., 2000; Crespo-Bellido et al., 2021), and its AV1 gene has 98.4% sequence similarity to that of EACMV. The AV1 gene encodes the coat protein (CP), the only viral protein that has been implicated in whitefly transmission (Briddon et al., 1990; Harrison et al., 2002; Pan et al., 2020). Hence, EACMCV is a good choice for studying the transmission of EACMV-like viruses.

Susceptible cassava cultivars (cv. Kibandameno and cv. 60444) were infected with EACMCV and ACMV by biolistic inoculation and used as source plants for transmission by SSA1-SG1 whiteflies, the predominant whitefly biotype in western Kenya. One bioreplicate used Kibandameno as the source plant and two bioreplicates used 60444 as the source plant. Whiteflies fed on source plants for a 48-h AAP and then were moved to virus-free recipient plants for a 48-h IAP. Viral titers in source plants and recipient plants were measured by qPCR. Source plant titers were above 10 million copies/ng of total DNA for the ACMV genome components and above 5 million for the EACMCV genome components just prior to their use for the transmission (Figure 5B). In the seven recipient plants, the ACMV titers were above 30 million viral copies/ng of total DNA, while EACMCV DNAs were not detectable above the healthy plant control at 28 days post-IAP. The ACMV DNA-A:B ratio in the recipient plants was 0.24, which was similar to the source plants. Sequencing read counts were consistent with the qPCR results (Figure 5C). Viral read counts mapping to ACMV-A and ACMV-B in the source and recipient plants were above 40,000. The source plants had over 50,000 reads mapping to EACMCV-A and EACMCV-B, but fewer than 50 reads were mapping to EACMV DNA components in the recipient plants. The results showed that ACMV but not EACMCV can establish a systemic infection when transmitted by whiteflies in this experiment. It is important to note that the whitefly colony used for the transmission studies was generated from *B. tabaci* SSA1-SG1 individuals collected from fields in western Kenya, and the results could differ for another whitefly biotype collected from a different region.

## 4. Discussion

Cassava mosaic disease is transmitted by vegetative propagation and whitefly transmission. There is strong evidence that increased whitefly transmission was a key contributing factor to the severity and extent of the East Africa CMD pandemic in the 1990s (Legg et al., 2006). In contrast, recent surveys have suggested that vegetative propagation now accounts for the majority of CMB transmission in Kenya, and whitefly transmission is more likely to occur in western Kenya than in the coastal region (Mwatuni et al., 2015). However, it is not known whether the transmission mode impacts viral species diversity and influences CMB species on a regional scale.

Very few studies have investigated the impact of vegetative propagation on CMD. A recent laboratory study showed that CMB genome sequence diversity increases across multiple rounds of vegetative propagation (Aimone et al., 2021b) but could not address effects on CMB species diversity because the studies were initiated using infectious clones. In contrast, the studies reported here used viral inocula from field-infected plants to compare species diversity at the time of collection of field-infected cassava in western and coastal Kenya and after 3 years of vegetative cutting in a greenhouse. Our results suggest that the maintenance in the greenhouse favors the transmission of EACMV-like viruses, while ACMV is preferentially transmitted by whiteflies under controlled laboratory conditions using source plants inoculated with infectious viral clones.

Species diversity was richer in the western samples than in the coastal samples at the time of collection (2015) (Figure 4). EACMV was present in all samples at the coast but not in all samples from western Kenya. In contrast, ACMV was only detected in the western region. There were also differences in the EACMV-like species found in the western (EACMKV and EACMV-Ug) and the coastal areas (EACMZV). The viruses detected in the field-collected samples are consistent with other field studies in Kenya (Bull et al., 2006; Mwatuni et al., 2015; Ombiro, 2016; Koima et al., 2018; Were et al., 2021). After 3 years of maintaining the plants by vegetative cutting in a greenhouse, EACMV-like viruses became prominent in all plants, with EACMKV and EACMV in western samples and EACMV and EACMZV in coastal samples. In contrast, EACMV-Ug, with a recombinant coat protein region originating from ACMV, did not persist through vegetative propagation. ACMV also could not be detected in the western samples after vegetative propagation with the exception of two plants that had very low read counts for the virus.

The emergence of EACMV in the greenhouse over time could reflect whitefly transmission even though the plants underwent a consistent insecticide spraying regime. However, we think rampant vector transmission is unlikely because we did not detect convergence between the EACMV sequences in the western and coastal samples, which would have occurred if viruses had moved between plants. Our whitefly transmission results (Figure 5) also argue against efficient plant-to-plant transmission of EACMV in the greenhouse. Instead, we propose that EACMV and ACMV respond differently to vegetative propagation because of the distinct kinetics of their infection processes in cassava. It is possible that particular isolates emerge in the greenhouse due to the different selection pressures seen in the greenhouse compared with the field, as has been seen with TYLCV (Sánchez-Campos et al., 2018). ACMV develops symptoms and accumulates to high levels quickly after inoculation but then titers decrease and the plant recovers from ACMV symptoms, while EACMV-like viruses establish infection more slowly and do not recover over time (Vanitharani et al., 2004; Patil and Fauquet, 2009). As a consequence, EACMV, more than ACMV, has the potential to be maintained and become the predominant virus during repeated cycles of cassava regrowth.

We observed examples of a virus occurring in a 2018 sample but not in its corresponding 2015 sample. This was seen exclusively for the western samples and involved in the detection of EACMV or EACMKV. Part of our difficulty in detecting specific EACMV-like viruses is that they can form reassortants and function together in co-infections (Bull et al., 2007; De Bruyn et al., 2012). Specifically, EACMV and EACMKV have the same iteron sequences and can form reassortants (Argüello-Astorga et al., 1994; Argüello-Astorga and Ruiz-Medrano, 2001). EACMV-like viruses have very similar sequences due to recombination (Lefeuvre and Moriones, 2015; Crespo-Bellido et al., 2021) of different CMB species. The sequences of EACMV and EACMKV are highly similar for a large portion of the DNA-A component, only diverging significantly in the AC1 and AC2 genes. Thus, determining which reads map to EACMV or EACMKV can be challenging when using short-read sequencing and reference-guided assembly. This is particularly problematic when a plant contains two related viruses with large differences in their genome copy numbers, making it very difficult to detect the less abundant virus. We addressed this issue, in part, by using *de novo* assembly, which does not rely on mapping to reference genomes, to detect low-abundance species. Our results illustrate the importance of using a combination of reference-guided assembly and *de novo* assembly for the accurate identification of highly similar viral species. However, horizontal transfer (by whiteflies) would be another explanation for the detection of a new species or a very distinct haplotype of the same species over the 3 years of vegetative cutting. We see only limited evidence of horizontal transfer of viruses (i.e., EACMV DNA-A in W1 in 2018 is closely related to the same in W18, Supplementary Figure S3A), but we observed more divergence among EACMV populations in 3 years than expected (Duffy and Holmes, 2009). Although there may be some confounding horizontal transfer of viruses in the experiment, our results indicate that EACMV-like viruses are favored by cycles of vegetative regrowth. The loss of ACMV and EACMV-Ug, which shares most of the ACMV coat protein that is essential for whitefly transmission (Briddon et al., 1990; Höfer et al., 1997; Harrison et al., 2002; Rana et al., 2016; Saurav et al., 2019; Pan et al., 2020) in the greenhouse, is consistent with a central role of vector transmission for maintenance of these viruses.

We hypothesize that the persistence of ACMV and EACMV-Ug in western Kenya is facilitated by vector transmission. This idea fits with trends observed in the East Africa CMD pandemic when high levels of ACMV and EACMV-Ug were accompanied by the emergence of a new super-abundant whitefly population (Colvin et al., 2004; Legg et al., 2006, 2014). It is also supported by the evidence from a 2015 field survey (Mwatuni et al., 2015) that found EACMV-like, ACMV, and EACMV-Ug viruses in western Kenya and symptoms indicative of both vegetative and whitefly transmission in infected plants. In contrast, the survey only detected EACMV and EACMZV and symptoms consistent with the vegetative transmission in coastal Kenya.

Whitefly density is likely not the only reason behind the differences in vector transmission between western and coastal Kenya. This idea is supported by the 2015 survey described above that showed that whitefly populations were high in both regions (Mwatuni et al., 2015). Whitefly diversity and differential ability to transmit viruses could be contributing factors to the difference in virus species by region and the incidence of vector transmission. Whiteflies are separated into genetic biotypes based on mtCOI sequences (Mugerwa et al., 2012, 2018; Manani et al., 2017). The SSA1-SG1 and SSA1-SG2 biotypes have been associated with severe CMD (Ndunguru et al., 2016; Aimone et al., 2021a). A recent study found SSA1-SG1, SSA1-SG2, and SSA2 biotypes in western Kenya and SSA2 and SSA1-SG3 biotypes in coastal Kenya (Munguti et al., 2021). Whitefly transmission is present in western Kenya, where SSA1-SG1 and SSA1-SG2 are present. A few fields with the SSA1-SG1 biotype have also been found in coastal Kenya in 2021, which may correlate with the first instance of AMCV in coastal Kenya (Munguti et al., 2021). There have not yet been comprehensive studies to show whether whitefly biotypes transmit CMBs or various CMB species differently, but other begomoviruses are known to be transmitted by different biotypes at different rates (Zhao et al., 2019; Chi et al., 2020; Fiallo-Olivé et al., 2020; Pan et al., 2020; Gautam et al., 2022). Other factors that could impact what CMBs are present and how they are transmitted include cassava cultivar differences by region, the adaptation to those improved varieties, and environmental factors.

These results also have implications for CMD management in East Africa. EACMV and EACMV-like viruses represent the greatest risk to the cassava seed systems that rely on vegetative propagation and ratooning to generate planting material for smallholder farmers (Ceballos et al., 2020). Regions with high pressure from SSA1-SG1 and SSA1-SG2 whiteflies are likely to be at greater risk from ACMV and EACMV-Ug. These observations can inform the models of CMD emergence and spread and help to develop better control methods.

## Data availability statement

The data presented in the study are deposited in the NCBI Sequence Read Archive repository, BioProject PRJNA950083.

## Author contributions

AD curated data, analyzed data, established analysis methodology, and wrote the manuscript. BMu and JM conceptualized the experiment, collected data, and established the methodology for vegetative transmission. JH helped conceptualize the vegetative experiments and completed some data analyses. YR and VL curated data and analyzed samples. WS, BMw, and MW conducted whitefly transmission experiments in Nairobi. PL curated data through library construction. LJ provided training and supervision for undergraduate trainees and developed the framework for the undergraduate research study with JA-I. GK and AJ developed the methodology, experimental conceptualization, and design of whitefly transmission experiments. SD assisted with bioinformatic analyses and manuscript editing. LH-B assisted with validation, manuscript preparation, supervision, and editing. EA organized the field collections and maintenance of plants in the greenhouse at JKUAT. IC assisted with bioinformatic analysis and resources. JA-I conceptualized the experiments and methodology, organized and supervised the specimen collection in Kenya, and supervised data analysis and manuscript preparation. All authors contributed to the article and approved the submitted version.
