# Supplementary material for: Cassava begomovirus species diversity changes during plant vegetative cycles
Source: Front Microbiol. 2023 May 25;14:1163566. doi: 10.3389/fmicb.2023.1163566 (PMC10248227; doi:10.3389/fmicb.2023.1163566)
Supplement: Supplementary file 2 [file Data_Sheet_2.pdf]

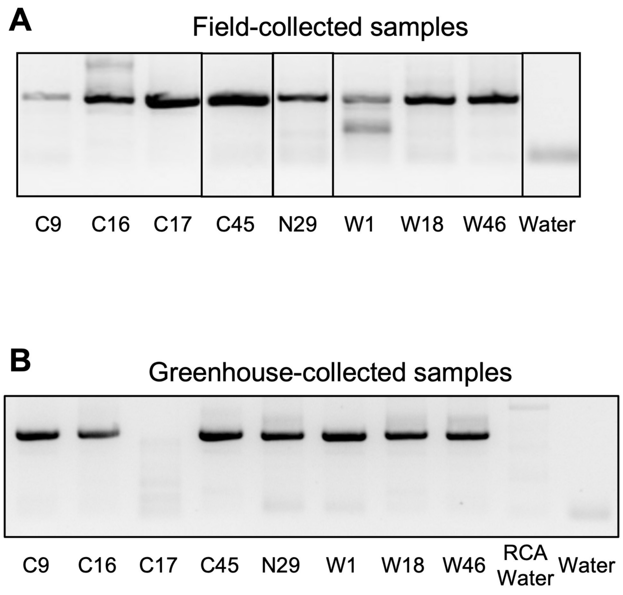

Figure S1. PCR for begomoviruses in field and greenhouse plants. Gels showing PCR products indicative of the presence of DNA-A components. **(A)** Bands amplified from field-collected samples of plants showing CMD symptoms. Four plants were sampled from western and from coastal Kenya in 2015. Images in panel A were cut from a larger gel image and arranged to show only the samples that were chosen for the sequencing experiment. **(B)** Bands amplified from the same plants that were sampled in 2018 after 3 years of greenhouse cutting.

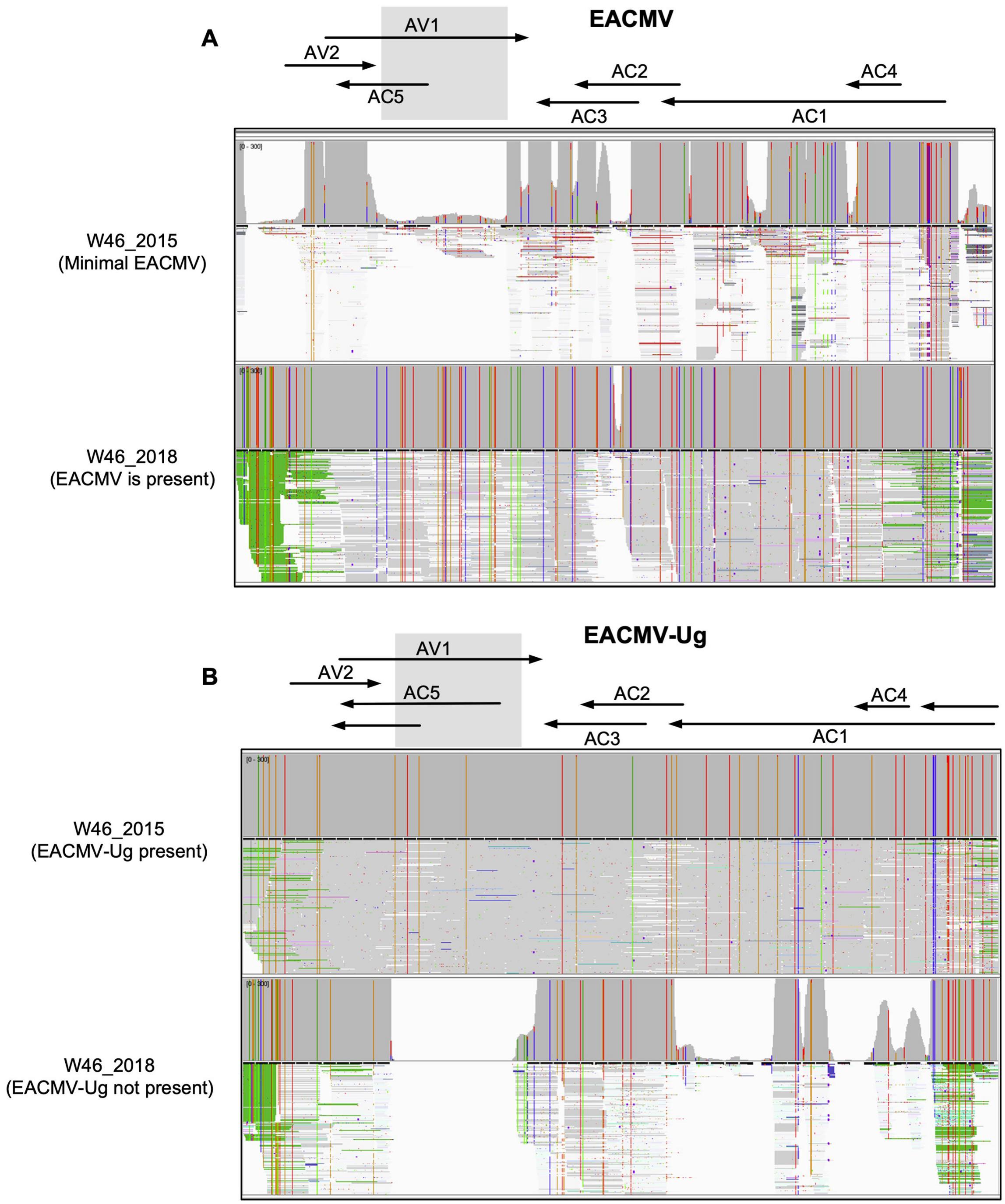

Figure S2. Comparison of EACMV and EACMV-Ug read coverage. Sequencing reads are visualized in an IGV genome browser showing differences in DNA-A coverage between samples with EACMV or the recombinant EACMV-Ug. EACMV and EACMV-Ug have different sequences in the AV1 gene (nt 544-1008) that distinguish the two variants. DNA-A components of EACMV and EACMV-Ug are circles that were linearized at the cleavage site for viral replication in the IGV images. **(A)** A diagram of the open reading frames for EACMV-A is shown at the top. The sample with EACMV-Ug (W46_2015) has spotty coverage when reads are mapped against EACMV and has especially low coverage in the recombinant region that distinguishes EACMV from EACMV-Ug. It does not reach the 300x continuous coverage cutoff. The sample with EACMV (W46_2018) shows full coverage reaching the 300x threshold across nearly the entire genome. **(B)** A diagram of the open reading frames for EACMV-Ug is shown at the top. The sample with EACMV-Ug (W46_2015) shows 300x coverage across the genome, while the sample with only EACMV shows spotty coverage and no reads in the recombinant region, indicating that EACMV is not present. The difference is that samples positive for EACVM-Ug will show a gap in the read coverage in the recombinant region when mapped against EACMV and samples positive for EACMV will show a gap in the read coverage in the recombinant region when mapped against EACMV-Ug. The difference in mapping helps identify which EACMV variant is present.

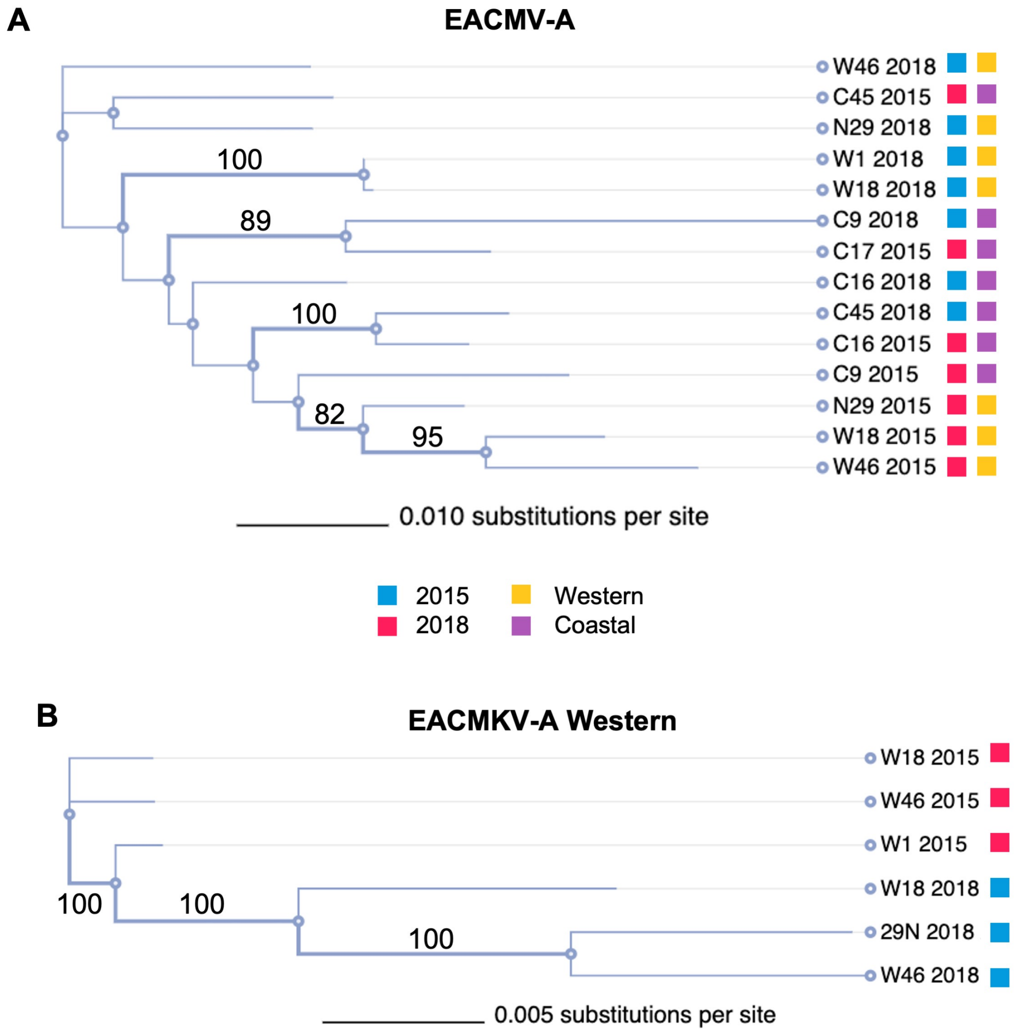

Figure S3. Phylogenetic trees of CMB sequences. **(A)** RAxML phylogenetic tree of EACMV-A sequences from both coastal and western areas. **(B)** 500 bootstrap RAxML tree of EACMKV DNA-A sequences from western Kenya. Bootstrap values (in percent) are shown above each branch. Only node bipartitions that were supported at a bootstrap support value ≥70% are shown. Branches are drawn to scale and the scale bar represents the number of substitutions per site.
