# Supplementary material for: Cassava begomovirus species diversity changes during plant vegetative cycles
Source: Front Microbiol. 2023 May 25;14:1163566. doi: 10.3389/fmicb.2023.1163566 (PMC10248227; doi:10.3389/fmicb.2023.1163566)
Supplement: Supplementary file 3 [file Data_Sheet_3.pdf]

Table S1. Library Statistics. Average read counts/library

Sample	Total reads	Host reads	Viral reads	Duplicate viral reads	Non-duplicate viral reads
Average	2.99E+07	1.88E+07	9.97E+06	8.25E+06	1.72E+06
C9_2015_A	3.80E+07	2.33E+07	1.59E+05	1.17E+05	4.21E+04
C9_2015_B	2.81E+07	2.85E+07	1.18E+05	9.16E+04	2.60E+04
C9_2018_A	2.32E+07	1.92E+07	4.88E+06	1.68E+06	3.19E+06
C9_2018_B	2.95E+07	2.45E+07	5.89E+06	1.76E+06	4.13E+06
C16_2015_A	2.17E+07	8.56E+06	1.67E+07	2.59E+06	1.41E+07
C16_2015_B	2.83E+07	1.12E+07	2.19E+07	2.94E+06	1.89E+07
C16_2018_A	3.93E+07	1.54E+07	3.04E+07	4.02E+06	2.64E+07
C16_2018_B	3.73E+07	1.55E+07	2.83E+07	3.87E+06	2.44E+07
C17_2015_A	3.09E+07	1.31E+07	2.29E+07	3.53E+06	1.94E+07
C17_2015_B	1.64E+07	6.42E+06	1.26E+07	2.53E+06	1.01E+07
C17_2018_A	2.70E+07	0.00E+00	3.40E+03	3.17E+03	2.26E+02
C17_2018_B	2.00E+07	2.60E+07	3.70E+03	3.49E+03	2.05E+02
C45_2015_A	3.07E+07	2.98E+07	1.84E+06	7.42E+05	1.10E+06
C45_2015_B	1.84E+07	1.77E+07	1.23E+06	5.49E+05	6.77E+05
C45_2018_A	2.89E+07	2.55E+07	3.92E+06	1.40E+06	2.52E+06
C45_2018_B	2.90E+07	2.57E+07	3.60E+06	1.28E+06	2.31E+06
W1_2015_A	2.90E+07	2.09E+07	2.90E+05	1.98E+05	9.21E+04
W1_2015_B	2.95E+07	2.25E+07	2.81E+05	1.96E+05	8.52E+04
W1_2018_A	5.28E+07	3.07E+07	3.02E+07	3.18E+06	2.71E+07
W1_2018_B	6.67E+07	4.13E+07	2.62E+07	3.36E+06	2.28E+07
W18_2015_A	3.84E+07	3.80E+07	3.02E+07	2.82E+05	3.00E+07
W18_2015_B	3.01E+07	2.98E+07	3.50E+05	2.21E+05	1.29E+05
W18_2018_A	3.24E+07	2.53E+07	8.69E+06	2.03E+06	6.66E+06
W18_2018_B	1.90E+07	1.42E+07	5.43E+06	1.62E+06	3.81E+06
W46_2015_A	2.35E+07	1.01E+07	1.70E+07	2.82E+06	1.42E+07
W46_2015_B	3.19E+07	1.30E+07	2.34E+07	3.34E+06	2.01E+07
W46_2018_A	2.35E+07	1.68E+07	8.05E+06	2.08E+06	5.97E+06
W46_2018_B	2.54E+07	1.76E+07	9.29E+06	2.23E+06	7.06E+06
N29_2015_A	2.00E+07	2.04E+07	9.61E+04	7.42E+04	2.19E+04
N29_2015_B	2.77E+07	2.78E+07	1.06E+05	7.75E+04	2.82E+04
N29_2018_A	3.28E+07	1.84E+07	1.92E+07	3.14E+06	1.60E+07
N29_2018_B	2.62E+07	1.45E+07	1.55E+07	2.85E+06	1.27E+07

Table S2. Contigs > 2 Kb generated by de novo assembly

Source Plant	Viral reference genome	Reference Accession	Length of Reference	Coverage	% Identity	Contig Name	Full length or Fragment	Length
W1_2015	EACMKV-A	AJ717570.1	2796	99	99.1	k141_79150	Full	2938
W1_2015	EACMKV-B	JF909217.1	2754	98	90.42	k141_78486	Full	2912
W1_2015	ACMV_B	HG530121.1	2724	99	98.21	k141_78461	Full	2727
W1_2015	ACMV_A	HE979766.1	2780	100	97.7	k141_39909	Full	2782
W1_2018	ACMV_A	J02057.1	2779	100	97.81	k141_96326	Full	2780
W1_2018	EACMV_B	AJ704936.1	2755	99	96.84	k141_7336	Full	2750
W1_2018	EACMV_A	AJ717551.1	2801	99	97.13	k141_3392	Partial	2721
W18_2015	EACMV_A	HG530113.1	2799	99	99.18	K141_47547	Full	2799
W18_2015	ACMV_B	KJ887692.1	2740	99	92.98	k141_110635	Full	2747
W18_2015	ACMV_A	AF126802.1	2782	100	98.8	k141_49839	Partial	2288
W18_2015	ACMV_B	HG530117.1	2724	100	99.05	k141_7549	Full	2722
W18_2018	EACMKV_A	AJ704971.1	2776	98	98.31	k141_34737	Full	2776
W18_2018	EACMV_A	MZ570970.1	2801	100	96.62	k141_39486	Partial	2269
W18_2018	EACMV-Ke_B	AJ704949.1	2757	100	98.02	k141_18601	Partial	1875
W18_2018	EACMV_A	AJ717571.1	2796	100	98.93	k141_145	Partial	1124
W46_2015	EACMV-Ug_B	AJ704961.1	2775	100	98.41	k141_3414	Full	2755
W46_2018	EACMV_A	AJ717549.1	2801	100	97.57	k141_8729	Full	2801
W46_2018	EACMKV_B	AJ704971.1	2776	100	97.98	k141_6065	Full	2775
29N_2018	EACMV_A	AJ717548.1	2801	99	98.89	k141_9088	Full	2800
29N_2018	EACMV_B	AJ704971.1	2776	100	97.84	k141_1595	Full	2775
9C_2015	EACMV_A	MZ570970.1	2801	99	97.39	K141_57579	Full	2801
9C_2015	EACMV_B	AJ704949.1	2757	100	98.61	k141_34402	Full	2942
9C_2018	EACMV_A	AJ717537.1	2801	99	98.53	k141_2781	Full	2801
16C_2018	EACMV_B	AJ704949.1	2757	99	96.55	k141_2779	Full	2754
17C_2015	EACMV_B	AJ704952.1	2754	100	98.08	k141_9973	Full	2754
17C_2018	EACMZV_A	AF422174.1	2785	99	98.6	k141_65584	Full	2785
17C_2018	EACMV_A	AJ717537.1	2801	100	98.31	k141_57574 +44123	Partial	2077
17C_2018	EACMV_B	AJ704949.1	2757	99	98.44	k141_55803 + 27071	Full	2761
W45_2015	EACMV_A	AJ717549.1	2801	100	97.68	k141_4743	Full	2800
W45_2015	EACMV_B	AJ704936.1	2755	100	96.73	k141_3803	Full	2755

Table S3. Comparison of consensus sequences between technical replicates[a]. Samples with sequence identity below 99.5% are highlighted in red.

Sample	Component	Percent	Nucleotide identity
9C_2015	EACMV_A	100%	2801/2801
9C_2015	EACMV_B	100%	2757/2757
9C_2018	EACMV_A	99.71%	2795/2803
9C_2018	EACMV_B	100%	2760/2760
16C_2015	EACMV_A	100%	2801/2801
16C_2015	EACMV_B	99.46%	2741/2756
16C_2015	EACMZV_A	99.93%	2783/2785
16C_2015	EACMZV_B	100%	2760/2760
16C_2018	EACMV_A	100%	2801/2801
16C_2018	EACMV_B	100%	2754/2754
16C_2018	EACMZV_A	99.93%	2783/2785
16C_2018	EACMZV_B	99.82%	2758/2763
17C_2015	EACMV_A	99.93%	2799/2801
17C_2015	EACMV_B	100%	2754/2754
17C_2015	EACMZV_A	100%	2785/2785
17C_2015	EACMZV_B	99.78%	2756/2762
45C_2015	EACMV_A	99.96%	2800/2801
45C_2015	EACMV_B	100%	2757/2757
45C_2018	EACMV_A	100%	2801/2801
45C_2018	EACMV_B	99.93	2753/2755
W1_2015	EACMKV_A	99.96%	2796/2797
W1_2015	ACMV_A	100%	2782/2782
W1_2015	ACMV_B	100%	2726/2726
W1_2018	EACMV_A	100%	2801/2801
W1_2018	EACMV_B	100%	2753/2753
W18_2015	ACMV_A	99.96%	2780/2781
W18_2015	ACMV_B	100%	2723/2723
W18_2015	EACMV-Ug_A	100%	2799/2799
W18_2015	EACMV-Ug_B	99.50%	2761/2775
W18_2015	EACMV_A	99.61%	2790/2801
W18_2018	ACMV_A	100%	2781/2781
W18_2018	EACMKV_A	97.28%	2722/2798
W18_2018	EACMKV_B	96.08%	2669/2778
W18_2018	EACMV_A	95.84%	2694/2811
W18_2018	EACMV_B	96.01%	2648/2758
W46_2015	ACMV_A	100%	2781/2781
W46_2015	ACMV_B	99.715	2717/2725
W46_2015	EACMV-Ug_A	100%	2798/2798
W46_2015	EACMV-Ug_B	100%	2775/2775
W46_2015	EACMV_A	99.75%	2794/2801

W46_2018	EACMV_A	99.18%	2780/2803
W46_2018	EACMV_B	100%	2756/2756
W46_2018	EACMKV_A	100%	2797/2797
W46_2018	EACMKV_B	99.93%	2773/2775
N29_2015	ACMV_A	100%	2779/2779
N29_2015	ACMV_B	99.82%	2740/2745
N29_2015	EACMV-Ug_A	99.64%	2789/2799
N29_2015	EACMV-Ug_B	100%	2775/2775
N29_2015	EACMV_A	99.75%	2794/2801
N29_2015	EACMV_B	99.24%	2733/2754
N29_2018	EACMKV_A	100%	2797/2797
N29_2018	EACMKV_B	99.93%	2772/2775
N29_2015	EACMV_A	99.96%	2800/2801
N29_2015	EACMV_B	100%	2696/2696

[a]Red shows samples with less than 99.5% identity
